# Optimal combination of feature selection and classification via local hyperplane based learning strategy

**DOI:** 10.1186/s12859-015-0629-6

**Published:** 2015-07-10

**Authors:** Xiaoping Cheng, Hongmin Cai, Yue Zhang, Bo Xu, Weifeng Su

**Affiliations:** 1 School of Computer Science& Engineering, South China University of Technology, Guangdong, China; 2 Electrical And Information College of Jinan University, Guangdong, China; 3 BNU-HKBU United International College, Hong Kong, China

**Keywords:** Feature weighting, Local hyperplane, Classification, Local learning, HKNN

## Abstract

**Background:**

Classifying cancers by gene selection is among the most important and challenging procedures in biomedicine. A major challenge is to design an effective method that eliminates irrelevant, redundant, or noisy genes from the classification, while retaining all of the highly discriminative genes.

**Results:**

We propose a gene selection method, called local hyperplane-based discriminant analysis (LHDA). LHDA adopts two central ideas. First, it uses a local approximation rather than global measurement; second, it embeds a recently reported classification model, K-Local Hyperplane Distance Nearest Neighbor(HKNN) classifier, into its discriminator. Through classification accuracy-based iterations, LHDA obtains the feature weight vector and finally extracts the optimal feature subset. The performance of the proposed method is evaluated in extensive experiments on synthetic and real microarray benchmark datasets. Eight classical feature selection methods, four classification models and two popular embedded learning schemes, including *k*-nearest neighbor (KNN), hyperplane *k*-nearest neighbor (HKNN), Support Vector Machine (SVM) and Random Forest are employed for comparisons.

**Conclusion:**

The proposed method yielded comparable to or superior performances to seven state-of-the-art models. The nice performance demonstrate the superiority of combining feature weighting with model learning into an unified framework to achieve the two tasks simultaneously.

**Electronic supplementary material:**

The online version of this article (doi:10.1186/s12859-015-0629-6) contains supplementary material, which is available to authorized users.

## Background

DNA microarray datasets can simultaneously determine the expression levels of thousands of genes [1]. For application purposes, these gene expression data must then be classified into various categories [2]. Together with classification methods, microarray technology has successfully guided clinical management decisions for individual patients, such as oncology [3, 4]. However, the sample size of the genetic dataset is usually much smaller than the number of genes, which extends into thousands or even tens of thousands [5]. Such limited availability of high-dimensional samples is particularly problematic for standard classification models. Feature selection technology, which seeks to eliminate irrelevant, redundant, and noisy genes while retaining all the highly discriminative genes, presents as an effective means of resolving this problem.

Various feature selection or dimensionality reduction methods have been proposed throughout the past decades. Among the most well-known unsupervised methods is Principal Component Analysis (PCA) [6] which preserves as much variance in the data as possible. Feature selection techniques can be broadly categorized into three groups; filter, wrapper and hybrid [7, 8]. The filter methods, such as Relief [9] and Mutual Information [10], identify feature subsets from the original feature set based on specific evaluation criteria that are independent of a learning algorithm. The wrapper methods use the classifier to evaluate the performance of each subset with a search algorithm. However, filter methods yield poor performance because they ignore classifier interactions, whereas wrapper methods are very computationally expensive. Hybrid methods [11] combine the advantages of both techniques to achieve nice learning performance with a predetermined learning algorithm and a reduced complexity.

Another type of feature selection model is discriminant analysis, which typically aims to minimize the margin between the inter-class and intra-class distances. For example, Fisher linear discriminant analysis (FLDA) searches for the embedding transformation that maximizes the between-class scatter while minimizing the within-class scatter. Recent research has concentrated on boosting the discriminative potential of these algorithm by exploiting the local data structure. Motivated by the great success of manifold local learning, researchers have proposed *localized* discriminant models such as locality preserving projections (LPP) [12], local discriminant embedding (LDE) [13], marginal Fisher analysis (MFA) [14] and locally linear discriminant analysis (LLDA) [15].

To fulfill data mining tasks, feature selection is usually followed by classification or clustering to reveal the intrinsic data structure. Although a few classification methods such as support vector machine (SVM) [16] could achieve the task of feature selection simultaneously, they are usually performed by separate algorithms. Such loose connection compromises the accuracy of the methods. Recently, some researchers have embedded the classifier into the discriminant analysis, and have reported remarkable experimental results. For example, a local mean-based nearest neighbor discriminant analysis (LM-NNDA) model was designed to construct classification rule in guiding the discriminator [17]. By optimizing a linear discriminant projection based on one nearest-neighbor (1-NN) classification scheme, the authors[18, 19] achieved both high classification accuracy and fast computational speed.

The present paper introduces a novel discriminant analysis model, named local hyperplane-based discriminant analysis (LHDA). This model optimizes the performance by combining feature selection with an effective classification scheme, namely, the K-Local Hyperplane Distance Nearest Neighbor (HKNN) classifier [20]. By minimizing the leave-one-out-cross-validation (LOOCV) error rate within the training phase, LHDA is shown to be optimally matched to the classifier of HKNN. The competitive performance of our method relative to established approaches is demonstrated in extensive experiments on synthetic and empirical datasets.

The advantages of our method are in three aspects: (1) Selection of the informative gene is conditioned on its linear combinations of similar peers, thus fully exploiting their joint discrimination power; (2) Incorporating the feature weighting within classifier learning process yields accurate feature weight and optimal classification performance simultaneously, thus fulfill the two important analysis task in a dynamic and tight way; (3) The superior performance of LHDA over its peers confirms that incorporation of interactions among similar genes in feature weighting estimation under local linear approximation, as well as relating the two tasks of feature selection and classification into an unified model not only revealing the informative genes, but also provides nice classification performance.

## Results and discussion

The performance of LHDA was evaluated in extensive experiments on various datasets. The first experiment was conducted on the famous Fermat’s Spiral synthetic data, which demonstrates the accuracy and robustness of LHDR in terms of feature weighting and classification, even when the data are highly degraded by noise. The second experiment was an empirical validation on 13 benchmark UCI datasets [21], which have low/median feature dimensions. The third experiment was conducted on practical 20 microarray datasets, which are characterized by large feature dimensions. Both the UCI datasets and microarray datasets were extensively tested in machine learning.

### Evaluation methods

Several state-of-art classification algorithm, including KNN [22], HKNN [20], SVM with linear (linear-SVM) and radical basis kernel (rbf-SVM) [16], were employed when comparing performance after feature selecting. Comparisons were also made against the discriminate analysis models LSDA [23], LDPP [18, 19] and LM-MNDA [17], and a well-known feature selection method called I-Relief [9, 24]. All four of these established models quantify the importance of features by incorporating local structures. In the final experiment, the algorithm was compared against eight standard feature selection methods combined with independent classification models. The performance of the classifiers was quantified by Leave-One-Out Cross-Validation (LOOCV), 10-fold cross validation (10-fold-CV) and inner Leave-One-Out Cross-Validation loop (inner LOOCV loop). In the LOOCV scheme, each sample in the dataset was predicted by building a model from the remaining samples and recording the accuracy of each model. In 10-fold-CV, the dataset was randomly divided into ten equally sized subsets. Nine of these subsets were used in the model construction and the remaining subset was used for prediction. In order to reduce the over-fitting problem as well as overcoming learning bias, an inner LOOCV scheme was used. Within the framework, each test sample is firstly removed from the dataset, resulting in a new training set. Then the whole learning process is carried out on the training set and tested on the left sample. The procedure is repeated for all tested samples and their averaged performance is calculated to quantify the performance of the learning model.

### Synthetic experiment on Fermat’s Spiral

**Fig. 1:**
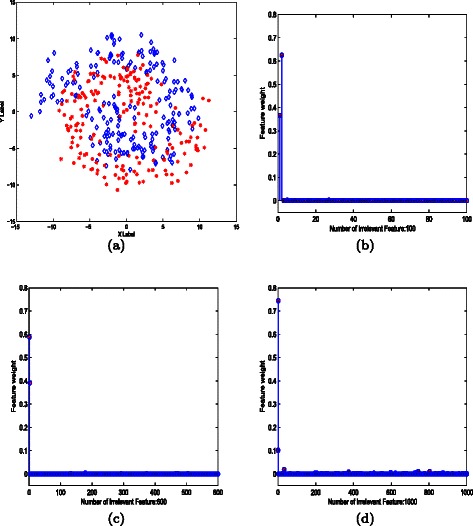
Experiments on feature weight estimation on Fermat’s Spiral. **a** Each class of 200 samples is labeled by a different color. To test the accuracy of feature weighting by LHDA, artificial noisy features of various dimensions (0 to 1000) were added to the dataset. The first two features completely determine the labels of the synthetic samples, while other features are redundant noises. These results are consistent with the data setting scheme. Estimated feature weights are plotted for noisy features of dimensions (**b**) 100; (**c**) 600; and (**d**) 1000

The synthetic dataset consists of two classes, each containing 200 samples. The labels of Fermat’s Spiral are completely determined by the first two features. The dataset distribution is shown in (Fig. 1a). Heuristically, one may observe that the label of a sample can be inferred easily from its local neighbors. Local information provides a more accurate classification assignment than global measurement based prediction (or classification), because the latter is sensitive to noise degradation. To test the stability and robustness of LHDA, irrelevant features were added to the Spiral. The irrelevant features were independently sampled from a zero-mean, unit-variance Gaussian distribution, and their dimensions were varied from 0 to 1000. The LHDA-based feature weights under noise of dimensions 100, 600 and 1000 are plotted in (Fig. 1b, c and d, respectively). Ideally, the labels of the Spiral should be completely determined by the first two features. Other features are presumably useless and should be assigned low weights. As shown in Fig. 1(b-d), most of the irrelevant features are assigned a weight of 0, demonstrating that the accuracy of feature selection by LHDA is robust to noise degradation.

**Fig. 2:**
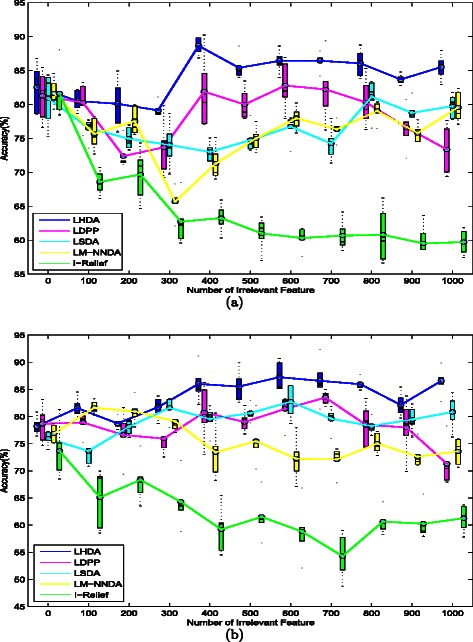
Performance of LHDA and various feature selection methods on Fermat’s Spiral problem with additional irrelevant features (dimensions ranging from 0 to 1000): (**a**) Experimental results of LOOCV; (**b**) Experimental results of 10-fold cross validation

The performance of LHDA was then compared with those of four feature selection techniques; LSDA [23], LM-NNDA [17], LDPP [18, 19] and I-Relief [9, 24]. These four techniques were selected because, like LHDA, they assign feature weights based on local data structure. Once the weights were obtained in each method, the classification performances were evaluated by applying the standard HKNN model to the feature-weighted spaces. To eliminate statistical variations, ten independent experiments were conducted on each dataset and the averaged classification accuracies were recorded. The numerical results of the 10-fold CV and LOOCV are summarized in Additional file 1: Table S1 and Table S2, respectively. For illustrative purposes, the differences between LHDA and its peers, evaluated by both CV schemes, are presented as boxplots in (Fig. 2a-b). Regardless of noise level, the classification accuracy of the HKNN classifier is higher when applied to LHDA than to the other feature selection methods. The average classification accuracy comes is 84.0 *%* in LOOCV and 83.7 *%* in 10-fold cross validation. The performances of the three discriminant analysis schemes, namely, LHDA, LM-NNDA and LSDA, remained stable as more irrelevant features were added, that of LDPP deteriorated when the number of irrelevant features exceeded 600. Under both CV schemes, the LHDA demonstrated superior performance to the other four methods in terms of the averaged classification accuracy (see Additional file 1: Table S1 and Table S2).

To evaluate the efficiency of the five methods, we recorded their time costs in obtaining feature subsets. As shown in Table 1, the speed of LHDA relatively is not high, which is faster than LM-NNDA. With no surprises, the filter based method of I-Relief and LSDA acheived the highest performance.

**Table 1:** Summary the speed of five local based methods

Irrelevant feature NO.	Cost time(s)				
	LHDA	LDPP	LSDA	LM-NNDA	I-Relief
100	18.82	2.09	0.05	57.86	3.23
200	40.24	2.63	0.07	134.36	7.72
300	87.96	3.00	0.11	632.91	11.83
400	139.13	3.31	0.25	487.89	30.71
500	203.28	3.60	0.24	493.70	35.03
600	285.81	3.58	0.35	560.68	61.91
700	366.59	5.45	0.34	651.41	73.77
800	505.73	6.05	0.32	758.55	83.78
900	655.34	6.58	0.39	972.84	95.71
1000	699.42	7.11	0.35	1201.89	108.58

### Experiments on UCI datasets

The second experiment was conducted on 13 datasets downloaded from the UCI Machine Learning Repository [21]. Most of the tested datasets have low-dimensional features and are widely used in variousclassification model evaluation. For each dataset, the aforementioned feature selection methods were firstly used to have the projections, which were then used to scale the raw datasets into the feature space. Four benchmark classification models, including KNN, HKNN, linear-SVM and rbf-SVM, were employed to evaluate the performance of five feature selection methods; LHDA, LDPP [18], LSDA [23], LM-NNDA [17], I-Relief [9, 24]. The results of 10-fold-CV are summarized in Additional file 1: Table S3. The top performers were I-Relief and LSDA coupled to the rbf-SVM classifier, with average accuracies of 88.09 *%* and 87.78 *%*, respectively. The performance of LHDA is only marginally below that of I-Relief and LSDA.

However, in the LOOCV evaluation, LHDA trumped the benchmark algorithms, achieving an average accuracy of 84.72 *%* (see Additional file 1: Table S4). This result is anticipated because our model is optimized to achieve minimization of the LOO errors. The second-best performer was LSDA combined with the classic rbf-SVM (average accuracy =84.70 *%*). When counting the win/loss/tie number of LHDA over the others, LHDA-HKNN obtained the best performances, which is higher than the others.

To further evaluate LHDA method, we conduct inner LOOCV loop testing on the 13 UCI dataset, the experimental results are summarized in Additional file 1: Table S5. The proposed model of LHDA, coupling with HKNN and rbf-SVM achieved the optimal and suboptimal performance in terms of averaged accuracy. If one counts the win/loss/tie number of LHDA over the others, the proposed LHDA also obtained remarkable performance.

### Experiments on microarray datasets

In the third experiment, we tested the performance of the proposed algorithm on 20 binary microarray datasets, which are characterized by large/huge feature dimensions. These datasets have been widely studied and all are related to human cancers such as leukemia, and tumors of the central nervous system, lung, and prostate [25]. The characteristics of the datasets are summarized in Table 2. All datasets were downloaded from http://epi.grants.cancer.gov/ and http://www.biolab.si/supp/bi-cancer/projections/index.htm, and were preprocessed by *t*-test with a 0.05 confidence level.

**Table 2:** Summary of the tested microarray datasets

Dataset	Gene No.	Sample No.
Adenocarcinoma	9868	76
Colon	2000	62
SRBCT	2038	83
GCM	16063	280
Leukemia	7129	72
Leukemia1	5327	72
Leukemia2	11225	72
Ovarian	15154	253
AML-prognosis	12625	58
Breast	4869	77
CML	12625	28
Gastric	7129	30
Medulloblastoma	2059	23
CNS	7129	34
Prostate1	12600	102
Prostate2	12625	88
Prostate3	12626	33
DLBCL	7129	77
Lung	12533	181
Lymphoma	2647	62

We hope to demonstrate that LHDA selects the highly informative and diagnostic genes from each dataset. To this end, we combined LHDA and the benchmark algorithms with various classification models and quantified the information conveyed by the selected genes by the classification accuracy. Similar to the second experiment, the projections were first obtained by four feature selection methods; LDPP [18], LSDA [23], LM-NNDA [17], I-Relief [24] and the proposed LHDA. In the subsequent classification experiments, the KNN, HKNN and SVM classifiers were applied to the feature-weighted space. Because the number of samples was limited, the performances were evaluated by the LOOCV scheme alone.

The experimental results are summarized in Additional file 1: Table S6. In majority cases, the best results were yielded by the proposed LHDA model. Indeed, the classification accuracy of LHDA was 100 *%* in 10 of the 20 datasets. LHDA was especially proficient at selecting genes implicated in adenocarcinoma and colon cancer, with respective classification accuracies of 98.68 *%* and 95.16 *%* in linear-SVM. Overall, LHDA tested by linear-SVM achieved remarkably high rankings in 11 out of 20 datasets. Moreover, the averaged accuracies after four classifiers for each dataset reflect the accuracy of feature weighting, shown in the last column for the five feature weighing method. The highest accuracy after the five methods on each dataset was highlighted in bold. One may note that the LHDA ranked in the top of eleven times among twenty datasets, demonstrating that the feature weighting obtained could quantify the intrinsic structures adequately.

As shown in the last row of Additional file 1: Table S6, the highest and second-highest average performance was achieved by LHDA coupled to linear-SVM and HKNN, respectively. The top five methods, in order of decreasing average accuracy, were LHDA with linear-SVM (97.82 *%*), LHDA-HKNN (96.95 *%*), LSDA-HKNN (96.74 *%*), LDPP with rbf-SVM (96.60 *%*), and LHDA with rbf-SVM (96.37 *%*). The accuracies of these five top-ranking combinations were quite close. The proposed method yielded the highest average accuracy, implying that the discriminative power of LHDA is at least as high as other state-of-the-art methods. In order to further evaluate the proposed method, confusion matrices of the classification results for the aformentioned feature selection methods were drawn, shown in Additional file 1: Table S7-S10.

### Comparison with standard feature selection methods

To further demonstrate the accuracy of feature weights obtained by the proposed LHDA, we compared it against eight baseline feature selection models, namely, information gain (IG), twoing rule (TR), Gini index (Gini), sum minority (SumM), sum of variances (SumV), max minority (MaxM), t-statistic (t-test) and one-dimensional support vector machine (OSVM). The algorithm codes for these eight schemes are available through RankGene at http://genomics10.bu.edu/yangsu/rankgene. The proposed LHDA was also compared with two state-of-the-art embedded methods, Support Vector Machine - Recursive Feature Elimination(SVM-RFE) [26] and Random Forest [27].

In the two experiments, informative gene subsets were first identified by each feature selection method, and were then evaluated by the four classification models, KNN, HKNN, linear-SVM and rbf-SVM. In the first experiment, the number of informative genes was set to equal to the number found by LHDA. This configuration enables a simple subjective comparison and allows us to investigate the discriminative power given a limited number of informative genes. The LOOCV accuracy is reported in Additional file 1: Table S11. The second row of this table states the number of informative genes found by LHDA. LHDA delivered superior average accuracy performance over the other tested methods, and significantly outperformed the second-most accurate method. Again, the highest and next-highest performance was achieved by LHDA coupled to two of the four classifiers.

To test the performance of the embedding models, the classical methods of SVM-RFE and Random Forest were employed for comparison. The experimental results are summarized in Additional file 1: Table S12. One may note that the performance of the three embedding methods are very close. The averaged accuracies of feature weighting after the three classifiers on each dataset were reported in the last column. It suggested that the LHDA ranked in the top of ten times among twenty datasets. When testing the feature weights obtained from LHDA by classification models of HKNN and linear-SVM, both of which achieved remarkably high rankings in 12 out of 20 datasets. In comparison, the linear SVM-RFE archived the second rank of 9 out of 20 datasets. Finally, the LHDA defeated the SVM-RFE by achieving slightly highest averaged performance when both of them are tested by linear-SVM, as shown in the last row of Additional file 1: Table S12.

The time cost of the three methods were reported in Table. 3. As it shown, the speed of LHDA was higher than that of SVM-RFE but lower than RF did.

**Table 3:** Summary the speed of three embedded methods

Irrelevant feature NO.	Cost time(s)		
	LHDA	SVM-RFE	RF
100	18.82	26.71	1.44
200	40.24	86.21	2.91
300	87.96	165.85	3.46
400	139.13	278.81	4.49
500	203.28	468.72	5.76
600	285.81	705.27	6.91
700	366.59	1010.30	8.69
800	505.73	1365.20	9.73
900	655.34	1833.64	11.90
1000	699.42	2029.50	11.41

## Conclusion

In this work, we proposed a new discriminant analysis model. The proposed LHDA uniquely incorporates both the feature weight and local structure to guide data classification. Optimal feature weights (in terms of LOO) are obtained by minimizing the penalized optimization problem. The proposed LHDA therefore achieves both accurate feature weight estimation and robust supervised classification simultaneously. In addition, LHDA preferentially selects the highly informative and discriminative features from datasets, boosting the performance of HKNN and other classification models. A numerical scheme for efficient minimization was developed, and the method was evaluated in extensive synthetic, median- and high-dimensional biomedical data. Four benchmark classification models and twelve widely recognized feature selection methods were employed for comparisons. The performance ability of LHDA was equal to or superior to other state-of-the-art methods, as demonstrated in rigorous quantitative analyses.

## Method

### Notation and problem description

Let ***x***_*i*_∈*R* ^*D*^ (*i*=1,2,…,*N*) be *D*-dimensional samples with associated class labels *y*_*i*_∈{1,2,…,*c*}, where *N* and *c* are the numbers of samples and classes, respectively. Let ***X*** be the matrix of all samples: ***X***=(***x***_1_,***x***_2_,…,***x***_*N*_). The distance |·| between two sample points ***p*** and ***q*** is defined by

$$ \left | \boldsymbol{p}-\boldsymbol{q} \right |=(\left | p_{1}-q_{1} \right |,\left | p_{2}-q_{2} \right |,\ldots,\left | p_{D}-q_{D} \right |) $$

Let ***w***=(*w*_1_,*w*_2_,…,*w*_*D*_), constrained by $\sum _{i=1}^{D} w_{i} = 1$, denote the importance of fea tures in ***X***. Then the Manhattan distance between two samples ***p*** and ***q***, scaled by the feature weighting vector ***w*** is given by:

$$ d(\boldsymbol{p},\boldsymbol{q})=\boldsymbol{w}^{T}\left | \boldsymbol{p}-\boldsymbol{q} \right |=\sum\limits_{i=1}^{D}w_{i}\left |p_{i}-q_{i} \right | $$

The purposes of this paper is to establish a model which achieves both the supervised classification for a new sample ***x*** and its feature weight estimation of ***w***. To achieve the goal, a local hyperplane based discriminant analysis model (LHDA) is proposed. The aim of LHDA is to optimize a classification model, namely, the feature-weighted hyperplane *k*-nearest neighborhood (FHKNN) model, within a feature-scaled space to simultaneously achieve the feature estimation and supervised classification. Therefore, LHDA consists of two steps, supervised classification via FHKNN and feature estimation through local learning. We shall describe the two phases individually.

### Feature weighted hyperplane KNN model (FHKNN)

The dimensionality of high-dimensional data is usually reduced by an appropriate technique prior to data processing. Mapping the data of interest into an embedded non-linear manifold within the higher-dimensional space has gained wide recognitions in machine learning[12, 15]. The local hyperplane approximation adopted in the present paper maintains the robustness of local linear embedding models. It assumes that sample structure is locally linear and therefore lies in a locally linear hyperplane.

Mathematically, the local hyperplane(with respect to class assignment) of an observed sample ***x*** is constructed by spanning its nearest neighbors, transformed into feature space by ***w***:

$$ {LH}_{c_j}\left(\boldsymbol{x}\Big|\boldsymbol{w}\right)=\left\{\boldsymbol{s}\kern1em \Big|\kern1em \boldsymbol{s}=\boldsymbol{H}\boldsymbol{\upalpha } ={\alpha}_1{\boldsymbol{h}}_1+{\alpha}_2{\boldsymbol{h}}_2+\dots +{\alpha}_k{\boldsymbol{h}}_k\right\} $$

where ***H*** is a *D*×*k* matrix composed of *k* nearest neighbors in the j-th class of the sample ***x***: ***H***={***h***_1_,***h***_2_,⋯,***h***_*k*_}, with ***h***_*i*_ being the *i*-th nearest neighbor of class *j*, *j*=1,2,…,*c*. The parameter **α**=(*α*_1_,…,*α*_*k*_)^*T*^ can be viewed as the spanning coefficients of the hyperplane, which can be estimated by minimizing the distance between the sample **x** and its feature mapped local hyperplane:

$$\begin{array}{@{}rcl@{}} \begin{aligned} J(\boldsymbol{\alpha}|\textit{\textbf{w}})=& \min_{\boldsymbol{\alpha}}\textit{\textbf{w}}^{T}\left | \textit{\textbf{x}}-\textit{H}\boldsymbol{\alpha} \right |  &\\ =& \min_{\boldsymbol{\alpha}}\textit{\textbf{w}}^{T}\left | \sum\limits_{i=1}^{k}\alpha_{i} \textit{\textbf{x}}-\sum\limits_{i=1}^{k}\alpha_{i} \textit{\textbf{h}}_{i} \right | &\\ =& \min_{\boldsymbol{\alpha}}\textit{\textbf{w}}^{T}\left | \sum\limits_{i=1}^{k}\alpha_{i} (\textit{\textbf{x}}-\textit{\textbf{h}}_{i}) \right | & \\ =&  \boldsymbol{\alpha}^{T} \textit{\textbf{z}} \\ \text{subject to} &\\ &\sum\limits_{i=1}^{k} \alpha_{i} =1,~ \boldsymbol{\alpha} \geq 0, \end{aligned} \end{array} $$

where the vector ***z***=(***w*** ^*T*^|***x***−***h***_1_|,***w*** ^*T*^|***x***−***h***_2_|,…,***w*** ^*T*^ |***x***−***h***_*k*_|).

The aforementioned optimization can be reformulated as an equivalent logistic regression problem:

(1) $$\begin{array}{@{}rcl@{}} \begin{aligned} &\max_{\boldsymbol{\alpha}}~ log(1+exp(-\boldsymbol{\alpha}^{T} \textit{\textbf{z}}))&\\ \text{Subject to} &\\ &\sum\limits_{i=1}^{k} \boldsymbol{\alpha}_{i} =1, \boldsymbol{\alpha} \geq 0. \end{aligned} \end{array} $$

In this new formulation, the parameter ***α*** is be solved in linear time. Mathematical details of the derivation are provided in Additional file 2. In the final step, the observed new sample is assigned a label *c* ^∗^ decided by the class that minimizes the distance between the sample and its hyperplanes:

$$c^{*} = \arg\min_{j} d(\mathbf{x},{LH}_{c_{j}}(\mathbf{x}|\mathbf{w})). $$

### Feature estimation though local hyperplane approximation

The aforementioned model assumes that the feature weight is known *prior*, which is infeasible in practice. To tackle this problem, we learn the optimal feature weight vector by minimizing the Leave-One-Out (LOO) error rate of the FHKNN classifier on the training set **X**. In this paper, we adopt the following error energy function:

(2) $$\begin{array}{@{}rcl@{}} \begin{aligned} \boldsymbol{J}(\boldsymbol{w})&=\min_{\boldsymbol{w}}\frac{1}{N}\sum\limits_{i=1}^{N}S(R({\boldsymbol{x}_{i}}))&\\ \text{Subject to} &\\ &\mathbf w > 0 \\ \end{aligned} \end{array} $$

where $R{(\textit {\textbf {x}})}=\frac {d(\textit {\textbf {x}}, {LH}_{\textit {NH}}(\textit {\textbf {x}}|\textit {\textbf {w}}))}{d(\textit {\textbf {x}},{LH}_{\textit {NM}}(\textit {\textbf {x}}|\textit {\textbf {w}}))}$. *L* *H*_*NH*_(***x***|***w***) and *L* *H*_*NM*_(***x***|***w***) are the local hyperplanes of the sample **x**, constructed from the sample’s two nearest neighbors within the feature scaled space, where one is from the same class (called *the nearest hit* or NH) and the other is from a different class (called *the nearest miss* or NM) [24, 25]. The function *S*(·) is a step function defined by:

$$S(x) = \left\{ \begin{array}{lr} 1 & : x \geq 1\\ 0 & : x <1 \end{array} \right. $$

Note that Eq. (2) minimizes the error between the sample and its local hyperplane rather than the error between the sample and its nearest neighbors. Such an approach ensures robustness from noisy samples. Similar techniques have been successfully applied in [24, 25]. As the step function is non-differentiable at discontinuous points, it is approximated by a Sigmoid function with slope *β*:

$$ S_{\beta}(z) = \frac{1}{1 + e^{\beta (1-z)}} $$

The derivative of *S*_*β*_(*z*) is given by

$$ {S}'_{\beta}(z) =\frac{{dS}_{\beta}(z)}{dz}= \frac{\beta e^{\beta(1-z)}}{(1 + e^{\beta(1-z)})^{2}} $$

This modification renders the objective function Eq. (2) differentiable; consequently, the corresponding minimization problem can be efficiently solved by standard numerical algorithms. The error function can be rewritten as

$$\begin{array}{@{}rcl@{}} \begin{aligned} \textit{\textbf{J}}(\textit{\textbf{w}})=& \min_{\textit{\textbf{w}}}\frac{1}{N}\sum\limits_{i=1}^{N}S_{\beta}(R(\textit{\textbf{x}}_{i}))&\\ \text{Subject to} &\\ &\textit{\textbf{w}} > 0 \\ \end{aligned} \end{array} $$

Sparseness of the feature vector is achieved by imposing a regularization *l*_1_ penalty [28]:

(3) $$\begin{array}{@{}rcl@{}} \begin{aligned} \textit{\textbf{J}}(\textit{\textbf{w}})&=\min_{\textit{\textbf{w}}}\frac{1}{N}\sum\limits_{i=1}^{N}S_{\beta}(R(\textit{\textbf{x}}_{i})) + \lambda \left \| \textit{\textbf{w}} \right \|_{1}&\\ \text{Subject to}&\\ &\textit{\textbf{w}} > 0 \end{aligned} \end{array} $$

where *λ* is a trade-off term that penalizes the sparsity of the feature vector.

Because the *l*_1_ penalty term is non-differentiable, it is difficult to solve directly. Let ***v*** ^2^=***w*** (note ***w*** is a nonnegative vector), and rewrite the first equation in Eq. (3) as

$$\begin{array}{@{}rcl@{}} \textit{\textbf{J}}(\textit{\textbf{v}})=\min_{\textit{\textbf{v}}}\frac{1}{N}\sum\limits_{i=1}^{N}S_{\beta}(R(\textit{\textbf{x}}_{i})) + \lambda \left \|\textit{\textbf{v}} \right \|_{2}^{2}. \end{array} $$

The derivative of **J**(**v**) with respect to **v** is

$$\begin{array}{*{20}l} \frac{\partial \textit{\textbf{J}}} {\partial \textit{\textbf{v}}} &=2 \lambda \textit{\textbf{v}}+ \frac{2}{N}\sum\limits_{i=1}^{N}{S}'_{\beta}(R{(\textit{\textbf{x}}_{i}})) R{(\textit{\textbf{x}}_{i})}\\ &\left[\frac{\left | \textit{\textbf{x}}_{i}-{LH}_{NH}(\textit{\textbf{x}}_{i}|\textit{\textbf{w}}) \right |}{d(\textit{\textbf{x}}_{i}, {LH}_{NH}(\textit{\textbf{x}}_{i}|\textit{\textbf{w}})}-\frac{\left | \textit{\textbf{x}}_{i}-{LH}_{NM}(\textit{\textbf{x}}_{i}|\textit{\textbf{w}}) \right |}{d(\textit{\textbf{x}}_{i}, {LH}_{NM}(\textit{\textbf{x}}_{i}|\textit{\textbf{w}})}\right] \otimes \textit{\textbf{v}} \end{array} $$

Let ***Υ***=(*γ*_1_,*γ*_2_,⋯,*γ*_*N*_) and ***G***=(***g***_1_,***g***_2_,⋯,***g***_*N*_), where

$$\begin{array}{@{}rcl@{}} \textit{\textbf{g}}_{i} &=& \frac{\left | \textit{\textbf{x}}_{i}-{LH}_{NH}(\textit{\textbf{x}}_{i}) \right |}{d(\textit{\textbf{x}}_{i}, {LH}_{NH}(\textit{\textbf{x}}_{i})}-\frac{\left | \textit{\textbf{x}}_{i}-{LH}_{NM}(\textit{\textbf{x}}_{i}) \right |}{d(\textit{\textbf{x}}_{i}, {LH}_{NM}(\textit{\textbf{x}}_{i})} \\ \gamma_{i} &=& {S}'_{\beta}(R{(\textit{\textbf{x}}_{i})}) R{(\textit{\textbf{x}}_{i})} \end{array} $$

Then, the derivative of **J**(**v**) can now be compactly written as

$$ \frac{\partial \textit{\textbf{J}}}{\partial \textit{\textbf{v}}}= 2 \lambda \textit{\textbf{v}} +\frac{2 \boldsymbol{\Upsilon}^{T} \textit{\textbf{G}}}{N}\otimes \textit{\textbf{v}} = (2 \lambda \mathbf{1} +\frac{2 \boldsymbol{\Upsilon}^{T} \textit{\textbf{G}}}{N})\otimes \textit{\textbf{v}} $$

where ⊗ is the Hadamard operator. The optimization problem can now be solved by iterating the following update equation:

$$ \textit{\textbf{v}}^{(t+1)}= \textit{\textbf{v}}^{(t)}-\eta \frac{\partial \textit{\textbf{J}}}{\partial \textit{\textbf{v}}} $$

where *η* is the step size. Ultimately, the feature weight is calculated as ***w***=***v*** ^2^.

**Fig. 3:**
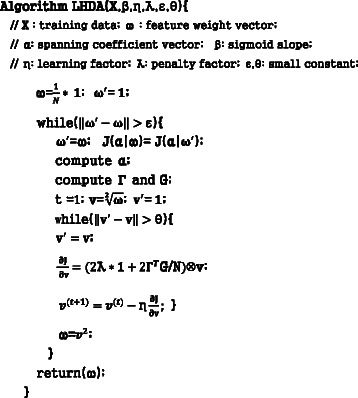
Algorithm of local hyperplane based discriminant analysis (LHDA)

The proposed algorithm is similar to the expectation maximization (EM) scheme. For a given feature weight ***w***, the spanning coefficients ***α*** and ***β*** for each sample are calculated, which are then used to correct the estimation of the feature weight ***w***. A pseudo code for the algorithm is presented in Fig. 3.

### Computational issues

The LHDA algorithm embeds the local data structure into the classification by minimizing its error in feature weighted space. It proceeds through two steps; approximating the local hyperplane of each sample and solving a minimizing problem to obtain the feature vector. The computational complexities of the hyperplane approximation and minimization steps in each iteration are *O*(*c* *k* *N* *D*) and *O*(*N* *D*), respectively. Here, *c* is the number of data’s class, *k* is the number of nearest neighbor we choose, *N* is the number of samples and *D* is the feature dimensionality.

## Availability of supporting data

The Matlab code used to tested on the Fermat Spirals and cancer microarray datasets are all available at http://pan.baidu.com/s/1hq8Bk2o.

## Additional files

Additional file 1
**Experimental results.** **Table S1.** Classification accuracies and standard deviations in the Spiral problem. Classification was performed by a standard HKNN scheme and evaluated by 10-fold cross validation criteria. The optimal and sub-optimal values on each tested data are highlighted in red and green. **Table S2.** Classification accuracies and standard deviations in the Spiral problem. Classification was performed by a standard HKNN scheme and evaluated by LOOCV criteria. The optimal and sub-optimal values on each tested data are highlighted in red and green. **Table S3.** 10-fold cross validation classification accuracies (%) for 13 UCI data sets processed by different dimensionality reduction techniques combined with different classifiers. The last row states the average classification accuracy. The optimal and next-optimal values for each tested dataset are highlighted in red and green, respectively. **Table S4.** LOOCV classification accuracies (%) for 13 UCI data sets processed by different dimensionality reduction techniques combined with different classifiers. The last row states the average classification accuracy. The optimal and next-optimal values for each tested dataset are highlighted in red and green, respectively. **Table S5.** Inner LOOCV loop classification accuracies (%) for 13 UCI data sets processed by different dimensionality reduction techniques combined with different classifiers. The last row states the average classification accuracy. The optimal and next-optimal values for each tested dataset are highlighted in red and green, respectively. **Table S6.** Classification accuracies (%) evaluated on 20 microarray datasets. The optimal and next-optimal values for each tested dataset are highlighted in red and green, respectively. The average performance of the proposed method is superior to that of the other methods. The averaged performance of the five feature weighting method on each dataset was calculated to evaluate their capabilities and the best values were highlighted in bold. **Table S7.** Confusion matrices of the classification results for KNN using different feature selection methods. **Table S8.** Confusion matrices of the classification results for HKNN using different feature selection methods. **Table S9.** Confusion matrices of the classification results for linear-SVM using different feature selection methods. **Table S10.** Confusion matrices of the classification results for rbf-SVM using different feature selection methods. **Table S11.** Performances of LHDA and 8 standard feature selection schemes (FSSs). The number of informative genes in all FSSs is the number determined by LHDA. The performance of the FSSs coupled to four classification models is evaluated by LOOCV. The optimal and second optimal accuracies (columnwise) of each tested dataset are highlighted in red and green, respectively. Where the dataset is not compatible with the method, the table entry has been left blank. **Table S12.** Classification accuracies (%) evaluated on 20 microarray datasets. The optimal and next-optimal values for each tested dataset are highlighted in red and green, respectively. The averaged performance of LHDA, Random Forest(RF) and SVM-RFE method on each dataset was calculated to evaluate their capabilities and the best values were highlighted in bold.

Additional file 2
**Numerical solution for FHKNN.**
