# Supplementary material for: Optimal combination of feature selection and classification via local hyperplane based learning strategy
Source: BMC Bioinformatics. 2015 Jul 10;16:219. doi: 10.1186/s12859-015-0629-6 (PMC4498526; doi:10.1186/s12859-015-0629-6)
Supplement: Additional file 1 — Experimental results. Table S1. Classification accuracies and standard deviations in the Spiral problem. Classification was performed by a standard HKNN scheme and evaluated by 10-fold cross validation criteria. The optimal and sub-optimal values on each tested data are highlighted in red and green. Table S2. Classification accuracies and standard deviations in the Spiral problem. Classification was performed by a standard HKNN scheme and evaluated by LOOCV criteria. The optimal and sub-optimal values on each tested data are highlighted in red and green. Table S3. 10-fold cross validation classification accuracies (%) for 13 UCI data sets processed by different dimensionality reduction techniques combined with different classifiers. The last row states the average classification accuracy. The optimal and next-optimal values for each tested dataset are highlighted in red and green, respectively. Table S4. LOOCV classification accuracies (%) for 13 UCI data sets processed by different dimensionality reduction techniques combined with different classifiers. The last row states the average classification accuracy. The optimal and next-optimal values for each tested dataset are highlighted in red and green, respectively. Table S5. Inner LOOCV loop classification accuracies (%) for 13 UCI data sets processed by different dimensionality reduction techniques combined with different classifiers. The last row states the average classification accuracy. The optimal and next-optimal values for each tested dataset are highlighted in red and green, respectively. Table S6. Classification accuracies (%) evaluated on 20 microarray datasets. The optimal and next-optimal values for each tested dataset are highlighted in red and green, respectively. The average performance of the proposed method is superior to that of the other methods. The averaged performance of the five feature weighting method on each dataset was calculated to evaluate their capabilities and the best values were hi [file 12859_2015_629_MOESM1_ESM.pdf]

Suppl. Table. 1 Classification accuracies and standard deviations in the Spiral problem. Classification was performed by a standard HKNN scheme and evaluated by 10-fold cross validation criteria. The optimal and sub-optimal values on each tested data are highlighted in red and green.

Methods	Number of Irrelevant Features											Average
	0	100	200	300	400	500	600	700	800	900	1000	
LHDA	78.2(1.7)	81.6(1.9)	78.7(1.0)	81.7(2.0)	86.0(3.3)	85.5(3.0)	87.3(2.8)	86.6(4.0)	86.0(1.1)	82.1(2.3)	86.6(2.6)	83.7
LDPP	78.7(3.5)	78.9(2.2)	76.6(4.5)	75.9(3.3)	80.8(5.3)	79.0(2.0)	81.4(2.9)	83.5(1.3)	78.5(4.0)	78.0(5.3)	71.3(4.2)	78.4
LSDA	75.9(1.3)	73.7(3.1)	78.1(1.5)	81.6(2.1)	79.5(2.5)	80.6(1.5)	82.7(3.1)	79.6(2.3)	78.2(1.4)	79.5(2.4)	80.8(3.2)	79.1
LM-NNDA	76.9(1.7)	81.6(1.3)	80.9(2.3)	79.1(2.4)	73.4(4.8)	75.5(4.2)	72.2(4.0)	72.1(2.5)	75.0(2.0)	72.6(2.4)	73.6(2.5)	75.7
I-Relief	73.7(5.0)	65.1(5.6)	68.3(4.1)	64.3(4.8)	59.2(4.4)	61.5(4.0)	58.8(5.3)	54.3(4.2)	60.7(2.3)	60.3(3.2)	61.3(2.6)	62.5

Suppl. Table. 2 Classification accuracies and standard deviations in the Spiral problem. Classification was performed by a standard HKNN scheme and evaluated by LOOCV criteria. The optimal and sub-optimal values on each tested data are highlighted in red and green.

Methods	Number of Irrelevant Features											Average
	0	100	200	300	400	500	600	700	800	900	1000	
LHDA	82.6(3.7)	80.5(1.6)	80.1(3.7)	79.1(1.1)	88.7(1.3)	85.4(1.9)	86.4(1.7)	86.4(1.4)	86.1(2.1)	83.7(0.8)	85.5(1.9)	84.0
LDPP	81.3(3.8)	80.3(3.1)	72.4(2.1)	73.7(4.1)	81.9(5.6)	80.0(2.4)	82.8(3.7)	82.2(4.9)	80.3(3.7)	76.6(3.2)	73.4(3.7)	78.6
LSDA	80.9(4.1)	76.5(1.7)	75.0(1.7)	74.0(3.4)	73.0(1.4)	74.7(2.1)	76.7(2.1)	74.2(2.5)	81.1(2.8)	78.8(2.0)	79.8(1.8)	76.8
LM-NNDA	81.6(2.1)	75.7(2.4)	77.6(2.1)	65.7(2.2)	71.3(2.6)	75.0(1.8)	78.0(1.7)	76.4(1.5)	79.1(1.5)	75.8(1.3)	79.6(2.4)	76.0
I-Relief	81.6(3.8)	68.6(1.8)	69.7(3.9)	62.7(3.5)	63.3(2.1)	61.0(2.5)	60.3(1.6)	60.7(2.3)	60.8(4.2)	59.5(3.3)	59.7(1.9)	64.4

Suppl. Table. 3 10-fold cross validation classification accuracies (%) for 13 UCI data sets processed by different dimensionality reduction techniques combined with different classifiers. The last row states the average classification accuracy. The optimal and next-optimal values for each tested dataset are highlighted in red and green, respectively.

Datasets	Method																			
	LHDA				LSDA				LM-NNDA				I-Rellief				LDPP			
	KNN	HKNN	linear-SVM	rbf-SVM	KNN	HKNN	linear-SVM	rbf-SVM	KNN	HKNN	linear-SVM	rbf-SVM	KNN	HKNN	linear-SVM	rbf-SVM	KNN	HKNN	linear-SVM	rbf-SVM
glass	71.54	76.75	64.39	76.12	67.23	67.25	64.53	78.27	72.57	75.86	63.91	82.46	73.94	75.89	64.27	79.72	66.97	69.61	64.38	77.48
cancer	78.89	80.81	76.33	80.18	76.83	77.11	80.81	81.13	77.25	75.08	80.23	80.47	80.57	77.56	80.73	80.78	77.56	77.59	80.31	78.26
wine	96.32	98.71	98.12	98.94	98.56	98.53	98.17	99.74	85.24	87.43	92.19	93.54	97.56	98.74	98.52	99.62	97.54	97.68	98.97	80.24
vote	96.86	97.18	96.72	97.59	97.27	95.68	96.67	97.06	91.67	93.83	97.75	97.46	95.22	94.54	96.38	96.98	95.87	95.30	96.48	80.45
zool	98.13	98.57	99.70	99.64	87.98	95.07	99.81	99.69	89.55	87.14	99.86	99.72	95.07	96.37	99.89	99.53	93.05	94.37	99.76	81.34
derm	95.47	96.85	98.87	99.27	97.03	95.93	98.95	99.59	79.63	81.76	95.65	96.43	97.86	97.89	99.37	99.74	98.67	98.76	99.57	82.78
Ionospere	89.47	93.89	90.10	93.45	85.35	91.24	89.65	95.17	90.76	92.86	87.45	94.72	87.56	90.73	89.42	96.57	92.14	92.67	90.46	84.67
pro	85.70	89.37	90.51	91.08	87.18	90.46	92.09	94.46	85.11	90.41	92.17	94.27	87.05	90.88	92.57	94.87	89.56	92.55	92.87	85.47
teach	47.23	66.97	65.78	65.85	52.24	60.04	66.37	74.08	61.24	62.43	66.75	78.07	57.57	52.13	67.52	75.25	51.27	60.23	66.12	86.26
Pima	72.56	73.77	77.97	77.29	74.42	73.21	77.94	77.52	75.34	74.89	77.82	77.51	75.45	72.70	77.07	77.86	74.58	74.31	77.56	87.41
bupa	59.46	67.36	68.64	73.55	62.57	63.51	69.07	72.14	60.95	60.59	69.57	71.86	63.07	64.45	66.58	70.39	60.77	64.96	66.71	88.45
heart	80.14	82.98	82.71	82.24	81.81	78.95	82.94	83.27	78.93	78.73	84.73	84.05	82.45	77.57	85.67	85.67	82.69	84.23	84.27	90.49
euk	80.07	84.10	81.43	83.93	79.51	85.73	83.28	89.10	78.06	84.62	84.14	89.45	76.17	83.75	83.24	88.24	74.23	75.80	83.07	89.76
Average	80.91	85.18	83.94	86.09	80.61	82.52	84.64	87.78	78.95	80.43	84.02	87.69	82.27	82.55	84.71	88.09	81.15	82.93	84.66	84.08
win/loss/tie	/	/	/	/	8/5/0	2/11/0	10/3/0	11/2/0	5/8/0	3/10/0	8/5/0	9/4/0	9/4/0	3/10/0	8/5/0	10/3/0	8/5/0	4/9/0	9/4/0	6/7/0

Suppl. Table. 4 LOOCV classification accuracies (%) for 13 UCI data sets processed by different dimensionality reduction techniques combined with different classifiers. The last row states the average classification accuracy. The optimal and next-optimal values for each tested dataset are highlighted in red and green, respectively.

Datasets	Method																			
	LHDA				LSDA				LM-NNDA				I-Rellief				LDPP			
	KNN	HKNN	linear-SVM	rbf-SVM	KNN	HKNN	linear-SVM	rbf-SVM	KNN	HKNN	linear-SVM	rbf-SVM	KNN	HKNN	linear-SVM	rbf-SVM	KNN	HKNN	linear-SVM	rbf-SVM
glass	71.03	76.64	67.29	67.29	64.02	69.16	69.63	71.50	74.77	73.36	68.22	71.50	74.77	76.64	65.42	70.56	70.09	76.17	64.95	67.76
cancer	77.78	81.31	79.80	79.80	75.25	77.27	79.29	79.29	76.26	76.77	76.26	81.31	80.30	77.78	81.31	81.31	77.27	80.81	82.83	87.37
wine	96.07	98.31	96.63	98.31	98.31	98.31	39.89	99.44	85.39	88.76	89.89	92.13	97.19	99.44	99.44	99.44	98.88	98.31	46.63	98.31
vote	96.55	97.41	96.98	96.98	96.98	95.69	75.86	96.98	91.38	93.10	94.40	96.98	95.69	93.97	95.69	96.98	95.69	95.26	83.62	96.55
zool	94.06	97.03	88.12	88.12	88.12	95.05	77.23	96.04	89.11	90.10	87.13	88.12	95.05	96.04	96.04	96.04	90.10	93.07	73.27	91.09
derm	96.45	97.27	93.72	93.72	96.72	95.63	32.79	95.90	79.51	80.87	80.33	85.79	97.27	97.81	97.54	97.54	98.36	98.63	81.69	98.91
Ionosphere	90.31	94.87	94.30	95.44	85.47	90.31	66.67	95.16	90.31	92.31	92.02	93.73	87.18	90.31	95.73	96.01	92.02	92.88	92.59	96.58
pro	83.05	87.36	86.56	86.56	88.16	90.97	74.52	90.77	85.66	90.67	73.02	90.67	87.76	91.07	90.77	90.17	89.57	91.78	83.05	91.27
teach	44.37	66.89	47.02	47.02	51.66	64.90	50.33	60.93	50.99	66.89	53.64	58.94	47.68	52.98	52.98	55.63	52.98	65.56	60.93	57.62
Pima	63.54	74.09	77.47	78.26	74.09	73.70	76.82	77.21	74.22	73.70	76.82	77.47	76.30	72.66	77.47	77.99	76.43	75.00	76.95	77.60
bupa	67.25	66.96	66.38	70.72	64.93	65.80	66.09	71.88	61.16	62.03	65.22	71.01	64.35	63.48	67.25	67.83	60.00	64.35	58.84	72.17
heart	80.74	82.96	83.33	83.33	81.85	79.63	59.26	82.59	80.00	78.52	75.56	84.07	82.59	79.26	79.63	85.56	83.70	84.44	61.48	84.44
euk	74.37	80.26	82.04	82.04	79.27	85.29	69.22	83.44	78.62	84.30	69.39	82.28	77.13	83.31	80.10	80.10	77.21	80.42	80.18	79.77
Average	79.66	84.72	81.51	82.12	80.37	83.21	64.43	84.70	78.26	80.88	77.07	82.62	81.79	82.67	83.03	84.24	81.72	84.36	72.85	84.57
win/loss/tie	/	/	/	/	8/5/0	2/10/1	2/11/0	8/4/1	5/7/1	2/10/1	2/11/0	7/4/2	10/3/0	4/8/1	8/4/1	9/3/1	8/5/0	5/7/1	2/11/0	9/3/1

Suppl. Table. 5 inner LOOCV loop classification accuracies (%) for 13 UCI data sets processed by different dimensionality reduction techniques combined with different classifiers. The last row states the average classification accuracy. The optimal and next-optimal values for each tested dataset are highlighted in red and green, respectively.

Datasets	Method																			
	LHDA				LSDA				LM-NNDA				I-Rellief				LDPP			
	KNN	HKNN	linear-SVM	rbf-SVM	KNN	HKNN	linear-SVM	rbf-SVM	KNN	HKNN	linear-SVM	rbf-SVM	KNN	HKNN	linear-SVM	rbf-SVM	KNN	HKNN	linear-SVM	rbf-SVM
glass	71.96	78.04	61.68	68.69	64.95	68.69	65.42	68.22	73.83	74.77	61.21	68.69	74.30	76.64	54.21	50.93	66.82	70.09	63.55	70.56
cancer	76.26	76.26	81.82	80.30	73.74	72.73	79.29	76.26	77.27	76.77	79.80	76.77	75.76	75.25	74.75	76.26	79.80	75.76	79.29	80.30
wine	96.07	96.63	98.88	98.31	97.19	97.19	97.19	98.31	85.96	87.08	91.01	84.27	97.19	99.44	97.75	96.07	96.63	95.51	97.19	98.31
vote	96.55	96.55	96.98	96.98	96.98	95.69	96.12	96.98	90.95	92.67	96.98	96.98	95.69	93.97	96.98	96.98	95.26	94.40	96.12	96.55
zool	94.06	95.05	95.05	94.06	90.10	95.05	95.05	87.13	87.13	91.09	90.10	88.12	92.08	96.04	88.12	83.17	91.09	92.08	94.06	92.08
derm	96.72	96.99	98.36	98.09	96.17	95.63	97.27	97.81	78.14	81.15	87.70	76.78	97.27	97.54	96.45	85.52	96.99	96.99	96.72	97.27
Ionosphere	87.18	93.73	88.32	95.16	85.19	88.03	88.60	94.30	90.31	92.31	88.03	91.17	86.89	89.46	89.17	85.47	89.46	91.74	86.32	92.88
pro	93.07	93.07	92.08	92.08	87.76	90.57	88.26	89.57	86.26	90.97	87.46	87.06	87.46	90.97	86.26	74.42	89.77	91.27	88.67	89.77
teach	54.30	66.23	52.98	56.29	50.33	64.24	57.62	56.29	49.67	66.23	54.30	51.66	52.98	56.29	51.66	43.71	54.30	66.23	50.99	54.97
Pima	73.70	73.05	77.21	77.47	74.09	73.31	77.08	75.39	74.09	73.57	76.82	76.04	75.13	72.01	77.60	77.47	73.31	72.01	76.69	74.87
bupa	66.67	68.41	66.96	71.88	64.64	66.09	68.41	69.57	61.16	61.74	68.41	69.28	65.22	64.35	61.45	64.93	64.06	64.93	65.22	65.51
heart	81.11	80.00	84.07	82.59	81.11	78.89	83.70	81.48	78.89	79.26	82.59	83.33	80.74	77.41	85.56	84.07	82.22	81.48	84.44	82.22
euk	77.21	83.44	71.86	73.05	78.57	85.25	70.83	72.11	77.96	84.30	70.79	74.87	76.76	83.35	66.25	61.10	76.60	80.68	69.02	69.96
Average	81.91	84.42	82.02	83.46	80.06	82.41	81.91	81.80	77.82	80.92	79.63	78.85	81.34	82.52	78.94	75.39	81.25	82.55	80.64	81.94
win/loss/tie	/	/	/	/	4/8/1	3/9/1	4/8/1	0/10/3	5/8/0	3/9/1	2/10/1	2/9/2	4/9/0	3/10/0	3/9/1	1/10/2	5/7/1	1/10/2	2/11/0	1/10/2

Suppl. Table. 6 Classification accuracies (%) evaluated on 20 microarray datasets. The optimal and next-optimal values for each tested dataset are highlighted in red and green, respectively. The average performance of the proposed method is superior to that of the other methods. The averaged performance of the five feature weighting method on each dataset was calculated to evaluate their capabilities and the best values were highlighted in bold.

Datasets	Method																								
	LHDA					LSDA					LM-NNDA					I-Rellief					LDPP				
	KNN	HKNN	linear-SVM	rbf-SVM	Aver.	KNN	HKNN	linear-SVM	rbf-SVM	Aver.	KNN	HKNN	linear-SVM	rbf-SVM	Aver.	KNN	HKNN	linear-SVM	rbf-SVM	Aver.	KNN	HKNN	linear-SVM	rbf-SVM	Aver.
Adenocarcinoma	86.84	93.42	98.68	90.79	**92.43**	88.16	94.74	89.47	90.79	90.79	85.53	89.47	90.79	84.21	87.50	85.53	89.47	85.53	93.42	88.49	85.53	93.42	90.79	94.74	91.12
Colon	83.87	90.32	95.16	93.55	90.73	87.10	87.10	85.48	85.48	86.29	83.87	85.48	88.71	71.0	82.27	82.26	79.03	77.42	85.48	81.85	91.94	88.71	91.94	95.16	**91.94**
SRBCT	100	100	100	100	**100**	100	100	100	100	**100**	100	100	100	100	**100**	100	100	95.18	100	98.80	100	100	98.21	100	99.55
GCM	87.86	92.50	98.21	99.29	**94.47**	91.07	92.86	87.50	87.50	89.73	87.50	91.43	97.86	97.86	93.66	86.79	93.21	90.71	91.79	90.63	86.07	90.36	87.86	92.50	89.20
Leukemia	100	100	100	98.61	**99.65**	91.67	100	100	100	97.92	98.61	98.61	98.61	97.22	98.26	97.22	98.61	95.83	98.61	97.57	98.61	98.61	97.22	98.61	98.26
Leukemia1	94.44	97.22	97.22	97.22	96.53	97.22	97.22	97.22	97.22	97.22	95.83	97.22	97.22	97.22	96.87	95.83	95.83	86.11	97.22	93.75	100	100	98.61	95.83	**98.61**
Leukemia2	98.61	98.61	100	100	99.31	100	100	98.61	98.61	99.31	98.61	100	97.22	84.72	95.14	98.61	100	97.22	100	98.96	100	100	100	100	**100**
Ovarian	100	100	99.21	99.21	99.61	99.21	100	100	100	99.80	100	100	100	100	**100**	100	100	100	100	**100**	98.81	99.21	100	100	99.51
AML-prognosis	84.48	96.55	100	98.28	**94.83**	91.38	94.83	91.38	93.10	92.67	79.31	81.03	87.93	60.34	77.15	86.21	84.48	84.48	93.10	87.07	86.21	82.76	89.66	91.38	87.50
Breast	84.42	89.61	100	98.70	**93.18**	80.52	87.01	90.91	90.91	87.34	75.32	76.62	71.43	66.23	72.40	85.71	85.71	79.22	83.12	83.44	80.52	81.82	79.22	85.71	81.82
CML	100	100	96.43	96.43	**98.22**	96.43	96.43	96.43	96.43	96.43	96.43	96.43	96.43	89.29	94.65	96.43	96.43	96.43	96.43	96.43	96.43	96.43	96.43	96.43	96.43
Gastric	96.67	100	100	100	99.17	100	100	100	100	**100**	96.67	96.67	100	73.33	91.67	96.67	96.67	100	100	98.34	96.67	100	93.33	100	97.05
Medulloblastoma	91.30	100	95.65	91.30	**94.56**	73.91	95.65	86.96	86.96	85.87	82.61	86.96	78.26	97.06	86.22	82.61	86.96	78.26	86.96	83.70	86.96	91.30	95.65	100	93.48
CNS	88.24	100	100	91.18	94.68	85.29	100	88.24	88.24	90.44	85.29	94.12	91.18	94.12	91.18	91.18	91.18	73.53	91.18	86.77	88.26	97.06	97.06	100	**95.60**
Prostate1	96.08	96.08	97.06	98.04	**96.82**	90.91	94.12	97.06	97.06	94.79	91.18	92.16	95.10	100	94.61	93.14	94.12	92.16	95.10	93.63	95.10	93.14	94.12	93.14	93.88
Prostate2	93.18	97.73	94.32	94.32	**94.89**	88.64	95.45	98.86	94.32	94.32	77.27	76.14	87.50	71.60	78.13	87.50	88.64	86.36	87.50	87.50	90.91	87.50	87.50	93.18	89.77
Prostate3	93.94	100	100	100	98.49	96.97	100	96.97	96.97	97.73	81.82	100	100	90.91	93.18	87.88	100	100	100	96.97	100	100	100	100	**100**
DLBCL	83.12	87.01	100	98.70	92.21	100	100	81.82	81.82	90.91	96.10	97.40	97.40	79.22	92.53	96.10	97.40	97.40	100	**97.73**	97.40	97.40	98.70	97.40	**97.73**
Lung	98.90	100	85.08	98.90	95.72	99.45	99.45	100	100	**99.73**	98.90	98.90	82.87	82.87	90.89	95.03	95.58	97.24	98.90	96.69	100	100	99.45	99.45	**99.73**
Lymphoma	96.77	100	99.45	82.87	94.77	100	100	100	100	**100**	98.39	98.39	100	100	99.20	98.39	98.39	100	100	99.20	100	100	99.45	98.39	99.46
Average	92.94	96.95	97.82	96.37	**96.02**	92.90	96.74	94.35	94.27	94.56	90.46	92.85	92.93	86.86	90.77	92.15	93.59	90.65	94.94	92.83	93.97	94.89	94.76	96.60	95.05
win/loss/tie	/	/	/	/	/	11/8/1	4/8/8	4/10/6	4/10/6	5/13/2	3/11/6	2/14/4	2/13/5	5/13/2	4/15/1	6/10/4	3/14/3	3/14/2	4/7/9	4/16/0	9/9/2	3/11/6	3/12/5	7/7/6	7/13/0

Suppl. Table. 7 Confusion matrices of the classification results for KNN using different feature selection methods.

Datasets	Methods				
	LHDA	LSDA	LM-NNDA	I-Rellief	LDPP
Adenocarcinoma	64 0<br>10 2	64 0<br>9 3	64 0<br>11 1	64 0<br>11 1	64 0<br>11 1
Colon	14 8<br>2 38	22 0<br>8 32	18 4<br>6 34	16 6<br>5 35	20 2<br>3 37
SRBCT	25 0 0 0<br>0 11 0 0<br>0 0 18 0<br>0 0 0 29	25 0 0 0<br>0 11 0 0<br>0 0 18 0<br>0 0 0 29	25 0 0 0<br>0 11 0 0<br>0 0 18 0<br>0 0 0 29	25 0 0 0<br>0 11 0 0<br>0 0 18 0<br>0 0 0 29	25 0 0 0<br>0 11 0 0<br>0 0 18 0<br>0 0 0 29
GCM	181 9<br>25 65	185 5<br>20 70	179 11<br>24 66	179 11<br>26 64	179 11<br>26 64
Leukemia	47 0<br>0 25	47 0<br>6 19	47 0<br>1 24	47 0<br>2 23	47 0<br>1 24
Leukemia1	8 1 0<br>0 24 1<br>0 2 36	8 0 1<br>0 24 1<br>0 0 38	7 0 2<br>0 24 1<br>0 0 38	7 0 2<br>0 24 1<br>0 0 38	9 0 0<br>0 25 0<br>0 0 38
Leukemia2	24 0 0<br>0 20 0<br>0 1 27	24 0 0<br>0 20 0<br>0 0 28	23 1 0<br>0 20 0<br>0 0 28	23 1 0<br>0 20 0<br>0 0 28	24 0 0<br>0 20 0<br>0 0 28
Ovarian	91 0<br>0 162	89 2<br>0 162	91 0<br>0 162	91 0<br>0 162	88 3<br>0 162
AML-prognosis	24 4<br>5 25	28 0<br>5 25	21 7<br>5 25	24 4<br>4 26	25 3<br>5 25
Breast	26 7<br>5 39	22 11<br>4 40	21 12<br>7 37	25 8<br>3 41	24 9<br>6 38
CML	12 0<br>0 16	12 0<br>1 15	12 0<br>1 15	12 0<br>1 15	12 0<br>1 15
Gastric	7 1<br>0 22	8 0<br>0 22	7 1<br>0 22	7 1<br>0 22	7 1<br>0 22
Medulloblastoma	13 0<br>2 8	13 0<br>6 4	12 1<br>3 7	12 1<br>3 7	13 0<br>3 7
CNS	25 0<br>4 5	25 0<br>5 4	25 0<br>5 4	25 0<br>3 6	25 0<br>4 5
Prostate1	48 2<br>2 50	44 6<br>3 49	47 3<br>6 46	47 3<br>4 48	49 1<br>4 48
Prostate2	35 3<br>3 47	32 6<br>4 46	25 13<br>7 43	32 6<br>5 45	33 5<br>3 47
Prostate3	24 0<br>2 7	24 0<br>1 8	24 0<br>6 3	24 0<br>4 5	24 0<br>0 9
DLBCL	50 8<br>5 14	58 0<br>0 19	55 3<br>0 19	56 2<br>1 18	57 1<br>1 18
Lung	31 0<br>2 148	30 1<br>0 150	29 2<br>0 150	22 9<br>0 150	31 0<br>0 150
Lymphoma	41 1 0<br>0 9 0<br>1 0 10	42 0 0<br>0 9 0<br>0 0 11	41 0 1<br>0 9 0<br>0 0 11	41 0 1<br>0 9 0<br>0 0 11	42 0 0<br>0 9 0<br>0 0 11

Suppl. Table. 8 Confusion matrices of the classification results for HKNN using different feature selection methods.

Datasets	Methods				
	LHDA	LSDA	LM-NNDA	I-Rellief	LDPP
Adenocarcinoma	64 0<br>5 7	64 0<br>4 8	64 0<br>8 4	64 0<br>8 4	64 0<br>5 7
Colon	20 2<br>4 36	22 0<br>8 32	18 4<br>5 35	15 7<br>6 34	19 3<br>4 36
SRBCT	25 0 0 0<br>0 11 0 0<br>0 0 18 0<br>0 0 0 29	25 0 0 0<br>0 11 0 0<br>0 0 18 0<br>0 0 0 29	25 0 0 0<br>0 11 0 0<br>0 0 18 0<br>0 0 0 29	25 0 0 0<br>0 11 0 0<br>0 0 18 0<br>0 0 0 29	25 0 0 0<br>0 11 0 0<br>0 0 18 0<br>0 0 0 29
GCM	190 0<br>21 69	190 0<br>20 70	186 4<br>20 70	186 4<br>15 75	181 9<br>18 72
Leukemia	47 0<br>0 25	47 0<br>0 25	47 0<br>1 24	47 0<br>1 24	47 0<br>1 24
Leukemia1	9 0 0<br>0 24 1<br>0 1 37	8 0 1<br>0 24 1<br>0 0 38	8 0 1<br>0 24 1<br>0 0 38	7 0 2<br>0 24 1<br>0 0 38	9 0 0<br>0 25 0<br>0 0 38
Leukemia2	24 0 0<br>0 20 0<br>0 1 27	24 0 0<br>0 20 0<br>0 0 28	24 0 0<br>0 20 0<br>0 0 28	24 0 0<br>0 20 0<br>0 0 28	24 0 0<br>0 20 0<br>0 0 28
Ovarian	91 0<br>0 162	91 0<br>0 162	91 0<br>0 162	91 0<br>0 162	89 2<br>0 162
AML-prognosis	26 2<br>0 30	28 0<br>3 27	22 6<br>5 25	23 5<br>4 26	22 6<br>4 26
Breast	29 4<br>4 40	23 10<br>0 44	23 10<br>8 36	25 8<br>3 41	25 8<br>6 38
CML	12 0<br>0 16	12 0<br>1 15	12 0<br>1 15	12 0<br>1 15	12 0<br>1 15
Gastric	8 0<br>0 22	8 0<br>0 22	7 1<br>0 22	7 1<br>0 22	8 0<br>0 22
Medulloblastoma	13 0<br>0 10	13 0<br>1 9	12 1<br>2 8	12 1<br>2 8	13 0<br>2 8
CNS	25 0<br>0 9	25 0<br>0 9	25 0<br>2 7	25 0<br>3 6	25 0<br>1 8
Prostate1	48 2<br>2 50	47 3<br>3 49	47 3<br>5 47	47 3<br>3 49	47 3<br>4 48
Prostate2	37 1<br>1 49	36 2<br>2 48	25 13<br>8 42	32 6<br>4 46	30 8<br>3 47
Prostate3	24 0<br>0 9	24 0<br>0 9	24 0<br>0 9	24 0<br>0 9	24 0<br>0 9
DLBCL	52 6<br>4 15	58 0<br>0 19	57 1<br>1 18	56 2<br>0 19	57 1<br>1 18
Lung	31 0<br>0 150	30 1<br>0 150	29 2<br>0 150	24 7<br>1 149	31 0<br>0 150
Lymphoma	42 0 0<br>0 9 0<br>0 0 11	42 0 0<br>0 9 0<br>0 0 11	41 0 1<br>0 9 0<br>0 0 11	41 0 1<br>0 9 0<br>0 0 11	42 0 0<br>0 9 0<br>0 0 11

Suppl. Table. 9 Confusion matrices of the classification results for linear-SVM using different feature selection methods.

Datasets	Methods				
	LHDA	LSDA	LM-NNDA	I-Rellief	LDPP
Adenocarcinoma	64 0<br>1 11	64 0<br>8 4	63 1<br>6 6	63 1<br>6 6	63 1<br>6 6
Colon	20 2<br>1 39	18 4<br>5 35	19 3<br>4 36	14 8<br>6 34	20 2<br>3 37
SRBCT	25 0 0 0<br>0 11 0 0<br>0 0 18 0<br>0 0 0 29	25 0 0 0<br>0 11 0 0<br>0 0 18 0<br>0 0 0 29	25 0 0 0<br>0 11 0 0<br>0 0 18 0<br>0 0 0 29	23 0 2 0<br>0 11 0 0<br>0 0 18 0<br>0 0 2 27	25 0 0 0<br>0 10 1 0<br>0 0 18 0<br>0 0 0 29
GCM	190 0<br>5 85	179 11<br>24 66	185 5<br>2 88	185 5<br>21 69	181 9<br>25 65
Leukemia	47 0<br>0 25	47 0<br>0 25	47 0<br>1 24	46 1<br>2 23	46 1<br>1 24
Leukemia1	9 0 0<br>0 24 1<br>0 1 37	8 0 1<br>0 24 1<br>0 0 38	8 0 1<br>0 24 1<br>0 0 38	8 0 1<br>0 24 1<br>0 8 30	9 0 0<br>0 25 0<br>1 0 37
Leukemia2	24 0 0<br>0 20 0<br>0 0 28	23 1 0<br>0 20 0<br>0 0 28	24 0 0<br>0 19 1<br>0 1 27	24 0 0<br>0 19 1<br>0 1 27	24 0 0<br>0 20 0<br>0 0 28
Ovarian	89 2<br>0 162	91 0<br>0 162	91 0<br>0 162	91 0<br>0 162	91 0<br>0 162
AML-prognosis	28 0<br>0 30	28 0<br>5 25	26 2<br>5 25	23 5<br>4 26	28 0<br>6 24
Breast	33 0<br>0 44	26 7<br>0 44	23 10<br>12 32	23 10<br>6 38	23 10<br>6 38
CML	12 0<br>1 15	12 0<br>1 15	12 0<br>1 15	12 0<br>1 15	12 0<br>1 15
Gastric	8 0<br>0 22	8 0<br>0 22	8 0<br>0 22	8 0<br>0 22	6 2<br>0 22
Medulloblastoma	13 0<br>1 9	12 1<br>2 8	11 2<br>3 7	11 2<br>3 7	12 1<br>0 10
CNS	25 0<br>0 9	25 0<br>4 5	23 2<br>1 8	19 6<br>3 6	25 0<br>1 8
Prostate1	49 1<br>2 50	50 0<br>3 49	48 2<br>3 49	46 4<br>4 48	48 2<br>4 48
Prostate2	36 2<br>0 50	38 0<br>1 49	32 6<br>5 45	32 6<br>6 44	32 6<br>5 45
Prostate3	24 0<br>0 9	24 0<br>1 8	24 0<br>0 9	24 0<br>0 9	24 0<br>0 9
DLBCL	58 0<br>0 19	48 10<br>4 15	57 1<br>1 18	57 1<br>1 18	57 1<br>0 19
Lung	4 27<br>0 150	31 0<br>0 150	0 31<br>0 150	26 5<br>0 150	30 1<br>0 150
Lymphoma	41 0 1<br>0 9 0<br>0 0 11	42 0 0<br>0 9 0<br>0 0 11	42 0 0<br>0 9 0<br>0 0 11	42 0 0<br>0 9 0<br>0 0 11	41 0 1<br>0 9 0<br>0 0 11

Suppl. Table. 10 Confusion matrices of the classification results for rbf-SVM using different feature selection methods.

Datasets	Methods				
	LHDA	LSDA	LM-NNDA	I-Rellief	LDPP
Adenocarcinoma	63 1<br>6 6	63 1<br>6 6	64 0<br>12 0	64 0<br>5 7	64 0<br>4 8
Colon	20 2<br>2 38	18 4<br>5 35	15 7<br>10 30	18 4<br>5 35	20 2<br>1 39
SRBCT	25 0 0 0<br>0 11 0 0<br>0 0 18 0<br>0 0 0 29	25 0 0 0<br>0 11 0 0<br>0 0 18 0<br>0 0 0 29	25 0 0 0<br>0 11 0 0<br>0 0 18 0<br>0 0 0 29	25 0 0 0<br>0 11 0 0<br>0 0 18 0<br>0 0 0 29	25 0 0 0<br>0 11 0 0<br>0 0 18 0<br>0 0 0 29
GCM	190 0<br>2 88	179 11<br>24 66	185 5<br>2 88	185 5<br>18 72	190 0<br>21 69
Leukemia	47 0<br>0 25	47 0<br>0 25	47 0<br>2 23	47 0<br>1 24	47 0<br>1 24
Leukemia1	9 0 0<br>0 24 1<br>0 1 37	8 0 1<br>0 24 1<br>0 0 38	8 0 1<br>0 24 1<br>0 0 38	8 0 1<br>0 24 1<br>0 0 38	7 0 2<br>0 24 1<br>0 0 38
Leukemia2	24 0 0<br>0 20 0<br>0 0 28	23 1 0<br>0 20 0<br>0 0 28	13 0 11<br>0 20 0<br>0 0 28	24 0 0<br>0 20 0<br>0 0 28	24 0 0<br>0 20 0<br>0 0 28
Ovarian	89 2<br>0 162	91 0<br>0 162	91 0<br>0 162	91 0<br>0 162	91 0<br>0 162
AML-prognosis	27 1<br>0 30	24 4<br>0 30	5 23<br>0 30	24 4<br>0 30	28 0<br>5 25
Breast	32 1<br>0 44	26 7<br>0 44	7 26<br>0 44	30 3<br>10 34	25 8<br>3 41
CML	12 0<br>1 15	12 0<br>1 15	9 3<br>0 16	12 0<br>1 15	12 0<br>1 15
Gastric	8 0<br>0 22	8 0<br>0 22	0 8<br>0 22	8 0<br>0 22	8 0<br>0 22
Medulloblastoma	13 0<br>2 8	12 1<br>2 8	13 0<br>1 9	12 1<br>2 8	13 0<br>0 10
CNS	23 2<br>1 8	25 0<br>4 5	25 0<br>2 7	25 0<br>3 6	25 0<br>0 9
Prostate1	50 0<br>2 50	48 2<br>1 51	50 0<br>0 52	49 1<br>4 48	47 3<br>4 48
Prostate2	36 2<br>0 50	36 2<br>0 50	20 18<br>7 43	32 6<br>5 45	35 3<br>3 47
Prostate3	24 0<br>0 9	24 0<br>1 8	24 0<br>3 6	24 0<br>0 9	24 0<br>0 9
DLBCL	57 1<br>0 19	48 10<br>4 15	48 10<br>6 13	58 0<br>0 19	57 1<br>1 18
Lung	31 0<br>2 148	31 0<br>0 150	0 31<br>0 150	29 2<br>0 150	30 1<br>0 150
Lymphoma	38 3 1<br>0 9 0<br>0 7 4	42 0 0<br>2 7 0<br>0 0 11	42 0 0<br>0 9 0<br>0 0 11	42 0 0<br>0 9 0<br>0 0 11	41 0 1<br>0 9 0<br>0 0 11

Suppl. Table. 11 Performances of LHDA and 8 standard feature selection schemes (FSSs). The number of informative genes in all FSSs is the number determined by LHDA. The performance of the FSSs coupled to four classification models is evaluated by LOOCV. The optimal and second optimal accuracies (columnwise) of each tested dataset are highlighted in red and green, respectively. Where the dataset is not compatible with the method, the table entry has been left blank.

Classifier	FSS	Adenocarcinoma	AML-prognosis	Breast	CML	CNS	Colon	DLBCL	Gastric	GCM	Leukemia	Leukemia1	Leukemia2	Lung	Lymphoma	Medulloblastoma	Ovarian	Prostate1	Prostate2	Prostate3	SRBCT	Avg.
	No. Genes	8	29	6	27	7	7	9	11	9	25	5	5	10	12	9	28	30	26	12	5	
KNN	IG	92.1	86.2	72.7	96.4	88.2	85.5	89.6	100.0	100	95.8	94.4	87.5	98.9	98.4	73.9	98.0	92.2	85.2	100	86.7	91.1
	TR	85.5	91.4	77.9	96.4	88.2	85.5	89.6	100	100	95.8	93.1	91.7	98.3	96.8	73.9	98.0	94.1	81.8	100	83.1	91.1
	Gini	85.5	86.2	75.3	96.4	91.2	88.7	90.9	100	100	97.2	91.7	90.3	98.9	93.5	73.9	98.0	93.1	79.5	100	77.1	90.4
	SumM	92.1	86.2	72.7	96.4	88.2	85.5	89.6	100	100	95.8	93.1	91.7	98.9	93.5	73.9	98.0	92.2	85.2	100	85.5	90.9
	SumV	92.1	86.2	72.7	96.4	88.2	85.5	89.6	100	100	95.8	93.1	91.7	98.9	95.2	73.9	98.0	92.2	85.2	100	86.7	91.1
	MaxM	90.8	93.1	79.2	96.4	88.2	88.7	88.3	100	100	98.6	93.1	91.7	98.3	95.2	82.6	98.8	93.1	77.3	100	84.3	91.9
	t-test	90.8	82.8	75.3	96.4	88.2	82.3	96.1	100	100	95.8			98.9		91.3	98.8	93.1	80.7	100		92.1
	OSVM		91.4	71.4	96.4	88.2	85.5	90.9	100	100	95.8			98.9		78.3	98.0	91.2	84.1	100		91.5
	LHDA	86.8	84.5	84.4	100	88.2	83.9	83.1	96.7	87.9	100	94.4	98.6	98.9	96.8	91.3	100	96.1	93.2	93.9	100	92.9
HKNN	IG	93.4	89.7	75.3	96.4	88.2	83.9	90.9	100	100	97.2	94.4	87.5	98.9	96.8	87.0	98.0	92.2	84.1	100	89.2	92.2
	TR	92.1	89.7	79.2	96.4	88.2	82.3	88.3	100	100	97.2	95.8	87.5	98.9	96.8	69.6	98.0	95.1	81.8	100	84.3	91.1
	Gini	86.8	87.9	79.2	96.4	88.2	82.3	90.9	100	100	95.8	94.4	87.5	99.4	93.5	60.9	98.0	94.1	81.8	100	79.5	89.8
	SumM	93.4	89.7	75.3	96.4	88.2	83.9	90.9	100	100	97.2	94.4	88.9	98.9	95.2	87.0	98.0	92.2	84.1	100	89.2	92.1
	SumV	93.4	89.7	75.3	96.4	88.2	83.9	90.9	100	100	97.2	91.7	91.7	98.9	98.4	87.0	98.0	92.2	84.1	100	89.2	92.3
	MaxM	93.4	94.8	79.2	96.4	91.2	87.1	83.1	100	100	97.2	91.7	91.7	97.8	98.4	82.6	99.6	91.2	78.4	100	89.2	92.1
	t-test	93.4	79.3	79.2	96.4	91.2	80.6	96.1	100	100	94.4			98.9		87.0	97.6	92.2	79.5	100		91.5
	OSVM		91.4	71.4	96.4	85.3	82.3	92.2	100	100	97.2			98.9		82.6	98.8	93.1	84.1	100		91.9
	LHDA	93.4	96.6	89.6	100	100	90.3	87.0	100	92.5	100	97.2	98.6	100	100	100	100	96.1	97.7	100	100	97.0
linear-SVM	IG	96.1	87.9	74.0	96.4	88.2	87.1	89.6	100	100	97.2	97.2	93.1	99.4	98.4	91.3	98.8	96.1	86.4	100	86.7	93.2
	TR	94.7	86.2	77.9	96.4	91.2	83.9	89.6	100	100	98.6	94.4	97.2	98.9	95.2	73.9	99.2	97.1	81.8	100	91.6	92.4
	Gini	89.5	79.3	75.3	96.4	91.2	87.1	93.5	100	100	98.6	94.4	93.1	99.4	98.4	78.3	98.8	95.1	77.3	100	80.7	91.3
	SumM	96.1	87.9	74.0	96.4	88.2	87.1	90.9	100	100	97.2	94.4	97.2	99.4	98.4	82.6	98.4	96.1	86.4	100	86.7	92.9
	SumV	93.4	87.9	74.0	96.4	88.2	83.9	90.9	100	100	97.2	94.4	97.2	99.4	98.4	87.0	98.8	95.1	86.4	100	94.0	93.1
	MaxM	92.1	91.4	80.5	96.4	91.2	88.7	90.9	100	100	98.6	94.4	97.2	97.8	98.4	87.0	100	95.1	86.4	100	94.0	94.0
	t-test	92.1	89.7	79.2	96.4	88.2	88.7	97.4	100	100	98.6			99.4		95.7	98.4	95.1	85.2	100		94.3
	OSVM		96.6	79.2	96.4	85.3	87.1	93.5	100	100	98.6			99.4		91.3	98.8	96.1	86.4	100		93.3
	LHDA	98.7	95.2	100	98.2	100	97.2	100	99.2	100	100	96.4	100	95.7	100	97.1	94.3	100	100	85.1	99.5	97.8
rbf-SVM	IG	96.1	89.7	77.9	96.4	88.2	85.5	89.6	100	100	98.6	97.2	93.1	99.4	96.8	69.6	98.8	96.1	86.4	100	90.4	92.5
	TR	96.1	87.9	75.3	96.4	91.2	85.5	93.5	100	100	98.6	93.1	97.2	99.4	98.4	69.6	99.2	95.1	85.2	100	92.8	92.7
	Gini	89.5	82.8	80.5	96.4	91.2	88.7	94.8	100	100	95.8	91.7	88.9	100	98.4	82.6	99.2	93.1	79.5	100	84.3	91.9
	SumM	94.7	86.2	77.9	96.4	91.2	85.5	92.2	100	100	97.2	94.4	97.2	99.4	98.4	91.3	98.8	96.1	85.2	100	90.4	93.6
	SumV	97.4	89.7	79.2	96.4	88.2	83.9	92.2	100	100	98.6	93.1	98.6	99.4	98.4	91.3	98.4	96.1	86.4	100	96.4	94.2
	MaxM	92.1	89.7	75.3	96.4	91.2	85.5	90.9	100	100	100	95.8	100	98.3	98.4	91.3	99.6	94.1	83.0	100	95.2	93.8
	t-test	92.1	86.2	79.2	96.4	88.2	87.1	98.7	100	100	97.2			100		91.3	98.4	94.1	83.0	100		93.6
	OSVM		94.8	77.9	96.4	91.2	88.7	93.5	100	100	97.2			99.4		82.6	99.2	92.2	89.8	100		92.8
	LHDA	90.8	93.6	100	99.3	98.6	97.2	100	99.2	98.3	98.7	96.4	100	91.3	91.2	98.0	94.3	100	98.7	98.9	82.9	96.4

Suppl. Table. 12 Classification accuracies (%) evaluated on 20 microarray datasets. The optimal and next-optimal values for each tested dataset are highlighted in red and green, respectively. The averaged performance of LHDA, Random Forest(RF) and SVM-RFE method on each dataset was calculated to evaluate their capabilities and the best values were highlighted in bold.

Datasets	Method														
	LHDA					RF					SVM-RFE				
	KNN	HKNN	linear-SVM	rbf-SVM	Aver.	KNN	HKNN	linear-SVM	rbf-SVM	Aver.	KNN	HKNN	linear-SVM	rbf-SVM	Aver.
Adenocarcinoma	86.84	93.42	98.68	90.79	92.43	86.84	93.42	97.37	97.37	**93.75**	84.21	90.79	96.05	98.68	92.43
Colon	83.87	90.32	95.16	93.55	**90.73**	87.10	83.87	85.48	88.71	86.29	88.71	85.48	93.55	88.71	89.11
SRBCT	100	100	100	100	**100**	93.98	93.98	97.59	95.18	95.18	98.80	100	100	100	99.70
GCM	87.86	92.50	98.21	99.29	**94.47**	87.86	90.36	87.14	90.71	89.02	85.00	86.79	88.93	88.57	87.32
Leukemia	100	100	100	98.61	99.65	94.44	94.44	94.44	93.06	94.10	100	100	100	100	**100**
Leukemia1	94.44	97.22	97.22	97.22	**96.53**	83.33	87.50	86.11	87.50	86.11	95.83	97.22	97.22	83.33	93.40
Leukemia2	98.61	98.61	100	100	**99.31**	88.89	91.67	93.06	93.06	91.67	98.61	100	100	94.44	98.26
Ovarian	100	100	99.21	99.21	**99.61**	98.81	97.63	98.42	98.81	98.42	98.81	99.21	100	99.21	99.31
AML-prognosis	84.48	96.55	100	98.28	94.83	87.93	86.21	87.93	87.93	87.50	96.55	98.28	98.28	96.43	**97.38**
Breast	84.42	89.61	100	98.70	**93.18**	80.52	81.82	89.61	87.01	84.74	90.91	87.01	84.42	100	90.58
CML	100	100	96.43	96.43	98.22	100	100	100	100	**100**	100	100	100	92.86	98.21
Gastric	96.67	100	100	100	99.17	100	100	100	100	**100**	93.33	100	100	94.12	96.86
Medulloblastoma	91.30	100	95.65	91.30	**94.56**	86.96	86.96	95.65	100	92.39	78.26	78.26	95.65	95.65	86.96
CNS	88.24	100	100	91.18	**94.86**	91.18	91.18	91.18	94.12	91.91	88.24	94.12	97.06	95.45	93.72
Prostate1	96.08	96.08	97.06	98.04	**96.82**	91.18	90.20	93.14	92.16	91.67	97.06	97.06	97.06	94.12	96.32
Prostate2	93.18	97.73	94.32	94.32	94.89	86.36	85.23	87.50	86.36	86.36	95.45	95.45	95.45	95.45	**95.45**
Prostate3	93.94	100	100	100	98.49	100	100	100	100	**100**	100	100	100	96.97	99.24
DLBCL	83.12	87.01	100	98.70	92.21	97.40	98.70	98.70	100	**98.70**	97.40	97.40	97.40	97.40	97.40
Lung	98.90	100	85.08	98.90	95.72	98.90	99.45	99.45	100	**99.45**	98.34	98.90	99.45	100	99.17
Lymphoma	96.77	100	99.45	82.87	94.77	93.55	95.16	96.77	96.77	95.56	98.39	98.39	100	100	**99.19**
Average	92.94	96.95	97.82	96.37	**96.02**	91.76	92.39	93.98	94.44	93.14	94.20	95.22	97.03	95.57	95.50
win/loss/tie	/	/	/	/	/	6/10/4	1/15/4	2/15/3	7/11/2	7/13/0	9/7/4	4/10/6	5/7/8	8/10/2	7/12/1
