# Supplementary material for: Optimal combination of feature selection and classification via local hyperplane based learning strategy
Source: BMC Bioinformatics. 2015 Jul 10;16:219. doi: 10.1186/s12859-015-0629-6 (PMC4498526; doi:10.1186/s12859-015-0629-6)
Supplement: Additional file 2 — Numerical solution for FHKNN. [file 12859_2015_629_MOESM2_ESM.pdf]

Numerical Solution for FHKNN

A sparse feature subset is obtained by adding a l_1 penalty term to the minimization functional Eq. (**??**); that is

$$\begin{aligned} \arg\max_{\boldsymbol{\alpha}} \quad & log(1 + exp(-\boldsymbol{\alpha}^T \boldsymbol{z})) - \delta \left\|\boldsymbol{\alpha}\right\|_1 \\ \text{subject to} \quad & \boldsymbol{\alpha} \geq 0. \end{aligned} \tag{S.1.6}$$

where δ is a trade-off coefficient that penalizes the sparseness of the features. Because the l_1 term $\|\boldsymbol{\alpha}\|_1$ is non-differentiable, the above maximization problem must be solved by direct methods such as the gradient descent method. To this end, we define $\boldsymbol{\beta}^2 = \boldsymbol{\alpha}$ and substitute it into Eq. (S.1.6) to obtain

$$\underset{\boldsymbol{\alpha}}{\text{maximization}} \quad log(1 + exp(\sum_{i=1}^{k} -\beta_i^2 z_i) - \delta \left\|\boldsymbol{\beta}\right\|_2^2$$

where $\boldsymbol{\beta} = (\beta_1, \beta_2, \cdots, \beta_k)$. This convex minimization problem is now directly solvable by the gradient descent method with the following update rule:

$$\boldsymbol{\beta}^{(t+1)} = \beta^{(t)} + \zeta(-\delta - \frac{exp(\sum_{i=1}^{k} -\beta_i^2 z_i)}{1 + exp(\sum_{i=1}^{k} -\beta_i^2 z_i)} \boldsymbol{z}) \otimes \boldsymbol{\beta}^{(t)}$$

where $\otimes$ is the Hadamard operator, and ζ is the learning rate determined by a standard line search.
